# Motor Skill Learning, Retention, and Control Deficits in Parkinson's Disease

**DOI:** 10.1371/journal.pone.0021669

**Published:** 2011-07-08

**Authors:** Lisa Katharina Pendt, Iris Reuter, Hermann Müller

**Affiliations:** 1 Department of Psychology and Sport Science, Justus-Liebig-University, Giessen, Germany; 2 Department of Neurology, Justus-Liebig-University, Giessen, Germany; Alexander Flemming Biomedical Sciences Research Center, Greece

## Abstract

Parkinson's disease, which affects the basal ganglia, is known to lead to various impairments of motor control. Since the basal ganglia have also been shown to be involved in learning processes, motor learning has frequently been investigated in this group of patients. However, results are still inconsistent, mainly due to skill levels and time scales of testing. To bridge across the time scale problem, the present study examined de novo skill learning over a long series of practice sessions that comprised early and late learning stages as well as retention. 19 non-demented, medicated, mild to moderate patients with Parkinson's disease and 19 healthy age and gender matched participants practiced a novel throwing task over five days in a virtual environment where timing of release was a critical element. Six patients and seven control participants came to an additional long-term retention testing after seven to nine months. Changes in task performance were analyzed by a method that differentiates between three components of motor learning prominent in different stages of learning: *Tolerance*, *Noise* and *Covariation*. In addition, kinematic analysis related the influence of skill levels as affected by the specific motor control deficits in Parkinson patients to the process of learning. As a result, patients showed similar learning in early and late stages compared to the control subjects. Differences occurred in short-term retention tests; patients' performance constantly decreased after breaks arising from poorer release timing. However, patients were able to overcome the initial timing problems within the course of each practice session and could further improve their throwing performance. Thus, results demonstrate the intact ability to learn a novel motor skill in non-demented, medicated patients with Parkinson's disease and indicate confounding effects of motor control deficits on retention performance.

## Introduction

Parkinson's disease (PD) is a neurodegenerative disease that mostly affects the basal ganglia (BG). Drug therapy or surgical treatment can provide relief from the predominant motor symptoms such as rigor, tremor, bradykinesia, hypokinesia, and hypometria. As there is currently no cure to the disease and the symptoms worsen with the progression of the disease, movement therapy is important to delay the loss of motor function. In that sense, practice, stabilization, and retention of movements, i.e. motor learning, play a major role in preserving quality of life in PD patients as long as possible. Since several lines of research provided evidence of BG involvement in motor learning [1]–[8], a considerable number of studies has tried to answer the question whether and how strong PD patients are impaired in learning motor skills. Although most studies have found impaired motor learning in PD patients [9]–[19], conclusions about characteristic learning deficits of PD patients are inconsistent; some find no impairments at all [20]–[23]. Besides methodological differences, operationalizations and specifics of the patient populations, different foci on distinct learning stages can account for discrepancies between studies because the BG are variably involved in the process of motor learning.

In de novo motor learning, one typically distinguishes early from late learning stages. Neuroimaging studies mostly use sequence learning tasks to investigate de novo motor learning and they suggest that in sequence learning tasks, the cerebellum is predominant over the BG in early sequence learning. This predominance decreases again with further practice [24]–[26], [3] and more BG activation is reported to occur later when performance reaches an asymptotic level [27], [26], [3], [28]. Furthermore, there is evidence from few studies that the BG are also important for the retention of learned motor skills [28]. Thus, in patients with BG dysfunctions, de novo motor learning can be differently affected depending on the learning stage. To our knowledge, there is no study specifically investigating motor learning of PD patients as a function of the learning stage. However, there are studies where, according to the study design, results suggest intact early learning for non-demented medicated patients [9], [20], [29], [10], [13], as well as differences between patients and the control group in later stages [10], [13]. There are only few studies investigating retention of performance in PD over at least 24 hours. Some suggest that retention might be impaired in PD patients [30], [12], [31] but others report intact retention [32], [13], [14], [23].

The inconsistencies in retention results can additionally be explained by confounding effects of motor deficits. Observations of such effects are frequently reported, although not systematically investigated. For instance, Swinnen et al. [33] showed that retention performance of PD patients can be influenced by motor control deficits. They had PD patients practice a bimanual figure drawing task over two days and reported performance decreases of the patients at the beginning of new practice sessions that could be ascribed to bradykinesia and hypometria. Similarly, Smiley-Oyen et al. [12] showed that PD patients had poorer performance in a movement scaling task (underscaling) at the beginning of practice and at a 24 h-retention test which might be related to hypometria. In agreement with this, other studies report a similar influence of motor control deficits on motor learning [10], [34], [19].

The purpose of this study is to analyze motor learning of a novel skill in medicated, mild to moderate, non-demented PD patients with respect to different learning stages, as well as the influence of symptomatic control deficits on the motor learning measure. Moreover, since retention of a practiced task is not well investigated in PD, we put a special emphasis on short- and long-term retention of performance, which is scrutinized by retention tests after varying time periods.

With respect to learning stages, we conceptually distinguish early from late learning in a novel motor skill as follows: Early learning is referred to using the concept of sensorimotor transformation. That is, a mapping between sensory inputs and motor commands needs to be established in order to solve a particular motor task [35]. This process occurs early in practice and improvements evolve relatively fast. With continued practice, the mapping is incrementally fine-tuned through constant comparison, selection and reinforcement of appropriate motor commands, as well as inhibition of unwanted, perturbing commands. We will call this fine-tuning stage. It is important, however, to mention that those phases do not develop in a discrete manner but rather overlap. Hence, they cannot be exactly temporally determined. Usually, stages are approximated from the amount of practice [12], evoked by experimental conditions [17], or specifically the fine-tuning stage is represented by reduction of variability [19]. We will use a method that exclusively and exhaustively identifies different components whose contribution to performance improvement varies across different learning stages (see Methods). Analysis of kinematic variables is used to highlight the influences of motor control deficits (see Methods).

Thus, focusing on de novo learning over a long series of practice sessions and based on the neuroimaging and patient literature, we can expect that mild to moderate, medicated, non-demented PD patients show similar improvements in early learning stages compared to healthy people (1), and that differences occur preferentially in later phases of fine-tuning (2). Not many studies investigated retention in PD, and results are inconsistent. Hence, we will not formulate an explicit expectation with respect to retention, but in reference to Swinnen et al. [33] and Smiley-Oyen et al. [12], we scrutinize whether retention deficits might be influenced by the typical parkinsonian motor control deficits (3).

## Materials and Methods

### Ethics Statement

All subjects were informed about the purpose of the study and gave written informed consent. The protocol was approved by the Ethical Review Board of the Justus-Liebig University, Giessen.

### Participants

19 patients with Parkinson's disease (PD) and 19 age and gender matched healthy subjects participated in the study. The PD patients were tested on medication and they all fulfilled the UK Brain Bank Criteria for the clinical diagnosis of PD. Six patients and seven control participants agreed to come to an additional retention testing after 7–9 months. Demographic and clinical information of the participants is given in Table 1 and 2. Exclusion criteria for both groups were any other neurological disease, orthopedic issues, and global cognitive deterioration as indicated by performance below 24 points on the German version of the Minimal Mental State Examination (MMSE) (originally [36]). All participants had normal or corrected to normal vision and they all were right-handed and used their right hand for the task. Patients had either bilateral or right unilateral symptoms.

**Table 1:** Demographic and clinical characteristics of PD and control group for the five-day practice sessions.

	PD (n = 19)			CG (n = 19)		
Variable	M	SD	Range	M	SD	Range
Age	63.9	8.8	45–77	64.8	10.1	44–80
Duration of PD (years)	6.8	5.7	1–20			
UPDRS motor score	26	9	11–41			
H&Y			1.5–3			
MMSE	27.6	2.3	22–30	28.6	1.3	26–30
Medication (mg): Levodopa (n = 19)	459.5	192	187.5–900			
Carbidopa (n = 19)	112	48	47–187.5			
Entacapon (n = 8)	771.4	335.2	200–1200			

UPDRS = Unified Parkinson's disease Rating Scale.

H&Y = Hoehn and Yahr rating scale.

MMSE = Mini Mental State Examination.

**Table 2:** Demographic and clinical characteristics of PD and control group for the long-term retention session.

	PD (n = 6)			CG (n = 7)		
Variable	M	SD	Range	M	SD	Range
Age	62	11.33	46–79	68	9.1	53–80
Duration of PD in years	5	0.9	4–6			
UPDRS motor score	24	6.7	16–44			
H&Y			2–3			
MMSE	28.8	1.5	26–30	28	1.3	26–30
Medication (mg):						
Levodopa (n = 6)	439.6	247.8	187.5–900			
Carbidopa (n = 6)	105.8	61.5	47–225			
Entacapon (n = 2)	900	424.3	600–1200			

### Task and Apparatus

**Figure 1:**
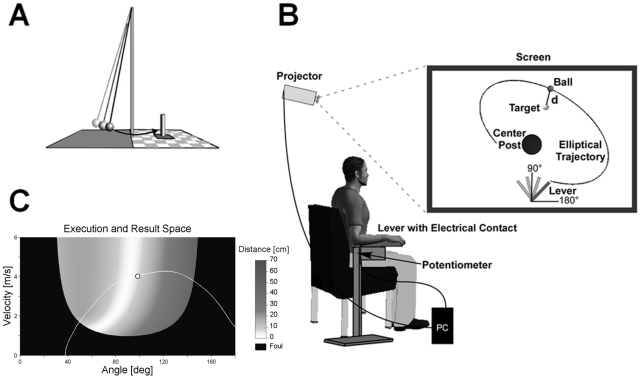
Experimental task. **A:** Sketch of the real Skittles task. A ball is suspended on a string and swings around the center post with the objective of knocking down the skittle at the opposite side. **B:** Experimental set-up. Participants operate a lever to throw the virtual ball on the screen in front of them with the goal to hit the target located behind the center post. The angular displacement of the participant's forearm is measured by a potentiometer and recorded by the computer. **C:** Execution and result space of the Skittles task. For each combination of the execution variables release angle and velocity the color codes the result variable, the minimal distance (d) of the resulting ball trajectory to the target (error). White denotes the solution manifold with zero-error solutions. Superimposed in white is the trajectory of one throw with its moment of release (white circle) at 98° and 4 m/s.

The experimental task was a semi-virtual throwing task called Skittles that has been used in other studies [37]–[39]. The idea of the task comes from a British pub game where a ball is suspended from a string attached to the tip of a vertical post. The player has to throw the ball around the post in order to knock down a target skittle on the other side (Fig. 1A). The movements of the participants in the experimental task were real, whereas the ball flight was virtual. Participants saw the work space of the task in two dimensions from a bird's eye view on the projection surface from which they sat approximately 2 m away. The post in the center of the work space was represented by a circle of 25 cm diameter at position x = 0, y = 0. A circular target of 5 cm radius was presented with its center 35 cm to the right and 100 cm above the center of the post. The virtual arm was represented as a solid bar of 40 cm length, fixed at one end (Fig. 1B). Sitting frontal to the projection screen, the participant rested his or her forearm on a metal arm (the manipulandum) with a plastic support padded with foam rubber. The horizontal manipulandum was fixed to a vertical support adjusted to a comfortable height for each participant and pivoted around an axle centered directly underneath the elbow joint. The elbow was fixed with a Velcro strap. Rotations of the arm were measured by a 5-turn potentiometer with a rate of 1000 samples/s. By touching an electrical switch at the free end of the metal arm, the virtual ball was attached to the virtual arm on the projection. Upon releasing the contact, the electrical current was disrupted and this accounted as trigger for releasing the virtual ball. Participants first closed the switch with their index finger, then rotated the forearm in an outward horizontal motion and simultaneously released the switch. The ball traversed on a trajectory initialized by the angle and velocity of the participant's arm at the moment of release. Both the movements of the arm and the simulated trajectory of the ball were displayed on the screen in real time. The ball's trajectory was determined by the simulated physics of the task and described an elliptic path around the pole [37]. The ball trajectory was not immediately intuitive to participants, and they had to learn the mapping between the real arm movements and the ball's trajectories in the projected work space. Hence, the task was new, even for participants with extensive throwing experience. The center post between arm and target impeded trivial solutions, i.e. releasing with zero velocity.

### Experimental Design and Procedure

Participants were instructed to throw the ball in a counter-clockwise direction around the center post in order to hit the target. The movement direction was clockwise, similar to performing a Frisbee backhand. After every 10 trials, a summed score, reflecting their performance (see Dependent Variables), was displayed on the screen. Participants were encouraged to keep their score as high as possible by achieving as many zero distance hits as possible and by avoiding collisions with the center post. The relation between execution variables, angle and velocity at the moment of release, and performance result is illustrated in Figure 1C.

After instruction and 30 test trials with a different target position on the first day, participants performed 200 throws per day. After each 100 throws, participants were given a short break; however, they could rest anytime in between if they wished. To assess short-term retention, participants performed five experimental sessions on five separate days, resulting in a total of 1000 trials. The first four sessions were scheduled on subsequent days; the fifth day was conducted after another five days. The subjects participating in the long-term retention session performed 200 additional trials 7–9 months after the practice period. Sessions were scheduled within one hour about the same time each day for individual participants.

### Dependent Variables

#### Assessing Performance Changes: the Score

The result measure was defined as the minimal distance (d) between the trajectory of the ball and the center of the target (Fig. 1B). This distance was converted to a score from 0 to 100 points where 100 points were achieved with a distance equal to zero. The score decreased linearly with increasing distance to the target and reached zero for a distance equal or higher than 50 cm (including center post hits).

#### Decomposing Performance Changes: the TNC-Method

**Figure 2:**
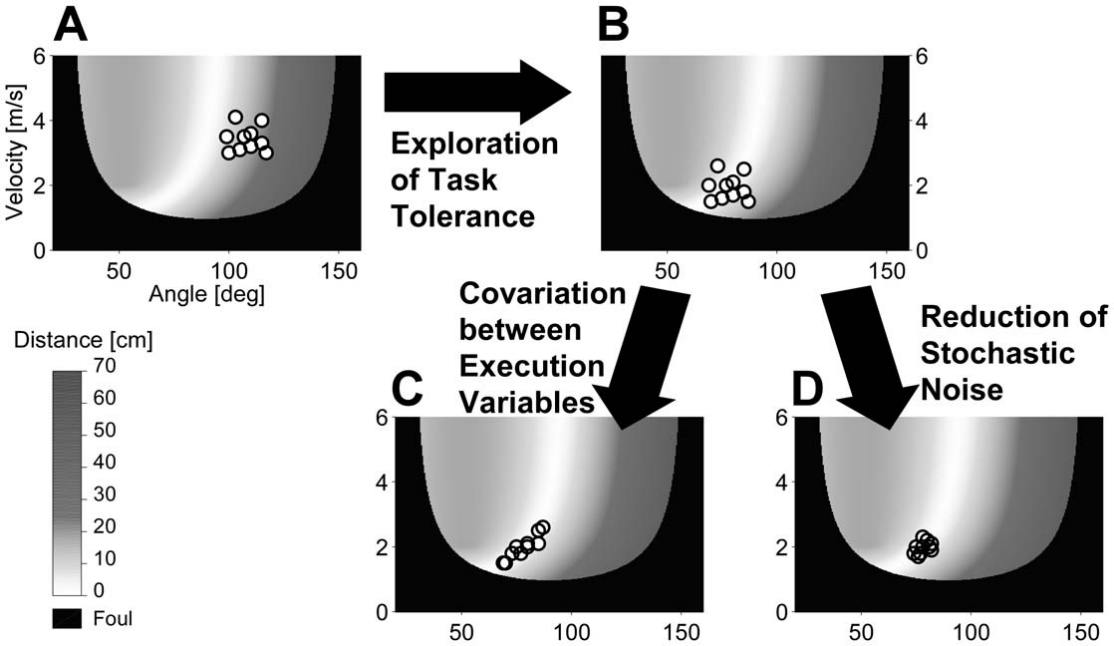
TNC components. Schematic Illustration of the three components contributing to performance change. **A:** A random set of 10 throws is plotted onto the execution and result space. The mean result of these trials, as indicated by the color coding, can be improved by shifting the cloud of trials to a more error tolerant area. That is, the mean performance of these 10 trials (with identical dispersion) would be better if execution variables were on average 80° and 1.9 m/s (**B**). This improvement can be achieved through exploration of the task space, searching for error (noise) tolerant areas. Hence, we call this component *Tolerance*. **C** and **D** show two additional options for performance improvement. Having found a tolerant solution (as in **B**), performance can be improved even further by co-varying the execution variables such that the trials align with the solution manifold (**C**). Note that the co-variated set of trials still has the same dispersion as the sets in **A** and **B**. Finally, reducing dispersion, or rather stochastic noise, can improve performance as well (**D**).

Motor learning in a goal-oriented task involves improvement in the accuracy of performance which can be broken down into three different, although not independent, components: exploitation of task tolerance, noise reduction, and covariation between the execution variables [37], [40] (see Fig. 2). These components prevail in different phases of the motor learning process. Since learning stages do not develop in a discrete manner but rather overlap, the prevailing learning stage is preferentially determined by the dominance of one component over the others. For instance, healthy subjects first exploit different combinations of execution variables when learning a goal-oriented task, i.e. they become familiar with the execution space and its mapping to results. Additionally, they seek combinations that are tolerant to noise, i.e. allowing a certain amount of motor variability and still solving the task. In Skittles, like in most motor tasks, this is possible since the task is redundant and hence offers an infinite set of solutions to achieve the same motor performance. This exploratory contribution to motor improvement is called exploitation of task tolerance, or *Tolerance (T)*. Müller and Sternad [37] could show that in over 70% of cases *T* had the highest contribution to performances changes in the first trials of practicing Skittles that decreased again with further practice. Hence, *Tolerance* is dominant in the early learning stage. With continued practice, performers fine tune their movements by reducing motor variability or noise and by covariation between execution variables (an error in one variable is directly compensated in the other variable). The dominance of these components called *Noise Reduction* (*N*) and *Covariation* (*C*) represents the fine-tuning stage.

In order to quantitatively determine the dominance of the components, they need to be compared. Müller and Sternad [37] developed a method that exclusively and exhaustively quantifies the contributions of the three components TNC by decomposing performance improvement. There exist two different approaches of the TNC method [37], [39] related to the same concept but addressing different aspects of it. Here, we apply the approach of Müller and Sternad [37]. In this approach, performance changes over a sequence of practice trials are extracted by comparing subsequent sets of trials. Each set is formed by the execution variables (here release angle and velocity) and has a mean performance result (here the score). The mean results of two subsequent sets A and B are compared and differences are decomposed in the TNC components by a stepwise reproduction of changes from set A to set B. Concretely, to analyze the data here, we combined practice trials in sets or blocks of 50. The contributions of the TNC components to changes in performance were quantified by calculating the difference in performance between two sequential blocks (ΔScore) and decomposing that difference into individual components. The contributions are non-overlapping and fully account for ΔScore: ΔScore = Δ*T*+Δ*N*+Δ*C*. The component with the highest contribution to a performance change from one block to the next is the prevailing one. Details about the calculation steps can be found in Müller and Sternad [37]. Note that when we speak of overlapping learning stages, we mean an incremental shift in dominance throughout practice between components, as indicated by different extents of contribution of the components to performance change, whereas the contributions themselves are non-overlapping.

#### Release Timing: Timeshift Measure

When adjacent angle velocity combinations of the moved manipulandum of several trials are plotted onto the execution and result space, they form a trajectory that performers produce preparing their throw (e.g. Fig. 1C). The release will lie on this trajectory and hence, performers seek to produce a movement trajectory that intersects the solution manifold (zero error solutions) in addition to optimally time their release at the intersection so that the ball hits the target. One can assume that after exploration of the task, performers find an adequate movement trajectory and keep it in a similar fashion over continued practice (as long as the task does not change). Timing of the release, on the other hand, is less stable. Due to its short time window, even subtle changes in neuronal processing can have essential consequences on the result. Hence, timing of the release represents an additional kinematic variable to analyze control processes in Skittles.

To analyze timing, the relative position of release points on the throwing trajectories of several subsequent trials were computed by a numerical procedure (see File S1 for more details). As a result, release timing of each trial is expressed with a measure in ms called *timeshift*, describing a relative time difference between release points on similar trajectories. *Timeshift* of one trial is positive when release of the trial is delayed relative to others and negative when release of the trial is early.

### Statistical Analyses

For each subject, score was averaged over blocks of 50 trials. Changes in score and TNC contributions across the 20 practice blocks and differences between days and groups were determined by a 2 (group)×5 (day)×4 (block) ANOVA with repeated measures. To address the influence of release control on retention performance, 50 trials before and 50 trials after a practice break were passed to the *timeshift* algorithm, resulting in a *timeshift* value for each of these 100 trials, expressing release timing of each trial relative to the others Thereafter, average *timeshift* of trials after rest was compared to trials before rest. To get a sensitive measure of change in *timeshift* after rest, five series of 10 post-rest trials each were averaged to create post-rest sets. Pre-rest sets consisted of the average of 50 pre-rest trials to provide a reliable reference for the comparison with the post-rest trials. The mean *timeshift* of the pre-rest set was then subtracted from the mean *timeshift* of each of the post-rest sets. These five resulting differences per break were compared between groups and the four practice breaks using a 2 (group)×4 (rest)×5 (series) ANOVA with repeated measures.

Results of the long-term retention session were analyzed with a 2 (group)×2 (block) ANOVA with repeated measures between the last practice block and the first long-term retention block. Besides statistical hypotheses testing, we used additional ANOVAs to validate data requirements or to exclude alternative explanations.

All statistical analyses were carried out using SPSS version 17.0. The level of significance was set at p<.05. If variance homogeneity was not given, the Greenhouse Geisser correction was applied to adjust the degrees of freedom and control for the violation.

## Results

We will illustrate the results in the following order: For all dependent variables, we first report the five practice days including short-term retention (24 h–5 days) before examining the long-term retention session after seven to nine months.

### Result Variable: Score

**Figure 3:**
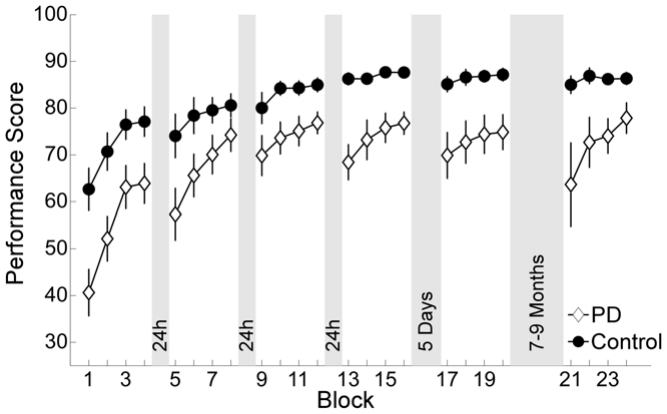
Performance scores over practice. Average performance over the course of five practice sessions and the retention session after 7–9 months for patients and the control group. Each session consisted of four blocks (50 trials). Note that there was a 24 hour break between the first four days and a five day break between the forth and the fifth day. Error bars denote the standard error of the mean.

Figure 3 displays average performance in the outcome measure score over blocks of 50 trials for both groups. Across the five practice days, the score increased in both groups (day: *F*(2.7,82.8) = 37.7, *p*<.001, *η_p_^2^* = .55), but the overall performance of the patients stayed below that of control group (group: *F*(1,31) = 13.7, *p*<.01, *η_p_^2^* = .31). Performance changes did not differ across days (day×group: *F*(2.7,82.8) = 1.7, *p* = .17, *η_p_^2^* = .05), i.e. PD patients and controls improved similarly over the five practice days. However, groups differed within days (block×group: *F*(1.9,58.4) = 3.3, *p*<.05, *η_p_^2^* = .10). This difference was due to a consistent drop in performance of the PD patients at the beginning of each new practice day: Initial performance of each day (as represented by the performance in the first block of every session) was lower than the performance in the last block of the previous day. Since overall performance of the patients was lower than performance of the control group, their performance increase within a day was steeper relative to controls. Although faster improvements on relatively low performance levels is typical in motor learning processes, it shows that patients were able to regain their previous performance and even exceed it, until performance leveled off on day four. An additional one way ANOVA with repeated measures did not reveal any significant differences between the performance drops, meaning the reduction was of similar size each day (*F*(2.1,27.1) = .27, *p* = .78, *η_p_^2^* = .02). Even on day five (after five days of rest) the decrease in performance was not worse compared to the other days.

To separately address initial improvement rate, an additional 2 (group) × 4 (block) ANOVA for only the first four blocks was conducted. No significant interaction effect was found (block×group: *F*(2.2,77.4) = 1.5, *p* = .22, *η_p_^2^* = .04), indicating that the improvement rate on the first practice day was similar between groups.

With respect to the long-term retention session, performance of the PD patients underwent, on average, a greater drop than the control group (Fig. 3), but this was not significant (*F*(1,11) = 2.7, *p* = .13, *η_p_^2^* = .20). Regarding the small group sizes in the long-term session, statistical power was too low to reach significance. Individual results showed a performance decrease only for two out of six PD patients (Table 3). The two deviant patients did not show differences in the general Parkinson motor rating scales. However, in the cognitive examination, scores were 2.75 (patient 16) and 4.75 (patient 25) points lower relative to the other four patients.

**Table 3:** Mean and SD of performance score of the PD patients (PD) and control subjects (C) for the long-term retention session.

	Block 20		Block 21	
	Mean	SD	Mean	SD
**PD 14**	73.6	16.9	73.5	25.1
**PD 15**	83.9	11.3	80.7	15.1
PD 16*	58.1*	24.7*	32.0*	24.7*
**PD 24**	84.6	18.0	85.7	9.0
PD 25*	86.5*	10.6*	41.6*	30.9*
**PD 26**	87.4	16.0	68.6	22.9
**C 3**	91.6	7.3	88.4	9.0
**C 6**	84.9	11.0	76.3	19.6
**C 8**	81.2	28.5	87.2	8.9
**C 9**	87.4	9.3	79.7	14.7
**C 11**	91.2	7.1	87.3	14.1
**C 17**	91.1	8.1	87.0	14.3
**C 22**	94.0	5.4	89.1	9.5

The last block of the fifth practice day and the first block of the long-term retention session are shown. Results of Patient 16 and 25, who showed performance decreases in long-term retention, are highlighted with an asterisk.

In summary, PD patients improved similarly well over five practice days compared to control subjects, whereas preservation of performance across breaks was lower. Long-term retention was not different from control subjects in four out of six patients.

### TNC Components

**Figure 4:**
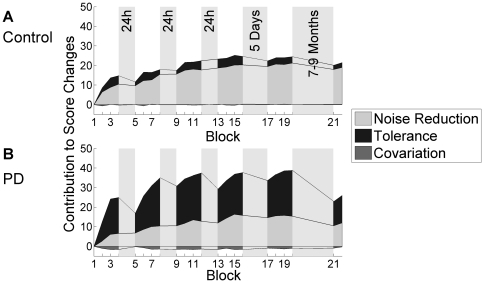
TNC-Contributions to performance changes over practice. Contributions of *T*, *N*, and *C* to performance changes over the course of the five practice sessions and the retention session for the control group (**A**) and PD patients (**B**).

We ascribed performance changes to the three different components with different time scales. *Tolerance* (*T*) is prominent in early learning and *Noise Reduction* (*N*) and *Covariation* (*C*) in the fine-tuning stage. Figure 4 depicts the contribution to performance changes over practice accounted for by each of the three components. Note that the measure is in score points and contributions are displayed in a cumulative fashion, i.e. contributions in one block are added to or subtracted from the previous block. Contributions are positive if the score increases and negative if the score decreases. Therewith, an increase in the area of a component represents an increase in its contribution to performance improvement and vice versa.

#### Tolerance

**Figure 5:**
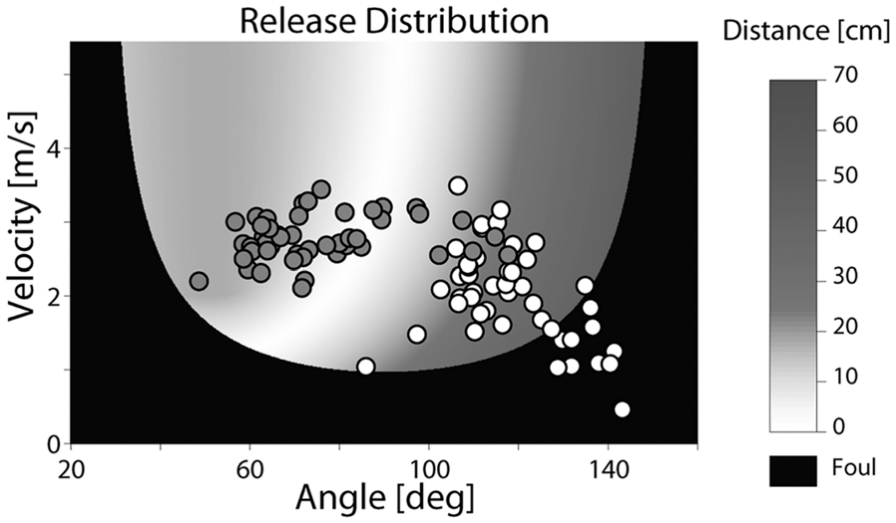
Initial execution areas and their influence on performance. Release and distance to target in execution space for a PD patient (white circles) and a control subject (gray circles) for the first 50 trials of practice.

Performance changes due to *T* are represented by the black area in Figure 4. Both groups used the component *T* to improve their performance (day: *F*(1.9,57) = 4.2, *p*<.01, *η_p_^2^* = .12; block: *F*(2,60) = 15.3, *p*<.001, *η_p_^2^* = .34). The contribution of *T* was significantly higher in the PD group than in the control group (group: *F*(1,30) = 7.1, *p*<.05, *η_p_^2^* = .19) due to the generally lower performance level of the patients. Patients started off in less tolerant areas than the healthy participants (Fig. 5). The same group×block interaction as in the score measure was observed (*F*(2.0,59.7) = 7.2, *p*<.01, *η_p_^2^* = .20). That is, the performance of the patients decreased because the contribution of *T* became negative in each first block of each new practice session. In other words, the patients performed worse in the beginning of each new session because, compared to the previous day, they were throwing in less tolerant areas. However, throughout a session they were able to find the better areas and hence increased their score again.

In the first long-term retention block, both groups showed a drop in *T*. Although this drop was larger in the PD group on average, there was no significant interaction effect (block×group: *F*(1,11) = 1.6, *p* = .23, *η_p_^2^* = .13) and the individual results revealed a significant negative contribution of *T*, similar to the score, only for patients 16 and 25 (Table 4).

**Table 4:** Contribution of Tolerance (*T*) to performance change from the last block of practice to the first block of the long-term retention session of patients (PD) and control subjects (C).

	Block 21		Block 21
	*T* Contribution		*T* Contribution
**PD 14**	12.8	**C 3**	−0.1
**PD 15**	−2.9	**C 6**	−7.2
PD 16*	−24.2*	**C 8**	2.2
**PD 24**	−1.0	**C 9**	−0.3
PD 25*	−45.2*	**C 11**	0.4
**PD 26**	−5.7	**C 17**	−3.2
		**C 22**	0.3

Results of Patient 16 and 25 are highlighted with an asterisk. Note that patient 14 considerably improved his performance through component *T* (by 12.8 points).

#### Reduction of Noise

The contribution to performance changes of *N* is illustrated by the light gray area in Figure 4. Both groups managed to enhance their score over the five practice days by reducing noise, i.e. the area of the *N* component increased (day: *F*(1.6, 48.1) = 27.2, *p*<.001, *η_p_^2^* = .48; block: *F*(2.1, 63.4) = 17.3, *p*<.001, *η_p_^2^* = .37). No significant group effect was found (*F*(1, 30) = 1.6, *p* = .22, *η_p_^2^* = .05). Furthermore, there was no significant interaction effect block × group (*F*(2.1, 63.4) = 1.2, *p* = .30, *η_p_^2^* = .04), indicating that the contribution of *N* was similar in both groups across all practice blocks.

For the long-term retention session, there was no significant block×group effect either (*F*(1, 11) = .47, *p* = .51, *η_p_^2^* = .04). Both groups showed a slight decrease in *N* contribution.

#### Covariation

*C* did not play a role in performance improvement for neither of the groups (Fig. 4 dark gray area). Neither group showed significant improvements due to *C*, and there were no significant differences between groups.

In summary, PD patients and control subjects used *Tolerance* to improve in Skittles. But in the patient group, *Tolerance* was also the component responsible for the performance decreases after rest. Contribution of *Noise Reduction* increased throughout practice in both groups. *Covariation* did not contribute to performance changes in neither group.

### Timing of Release: Timeshift

**Figure 6:**
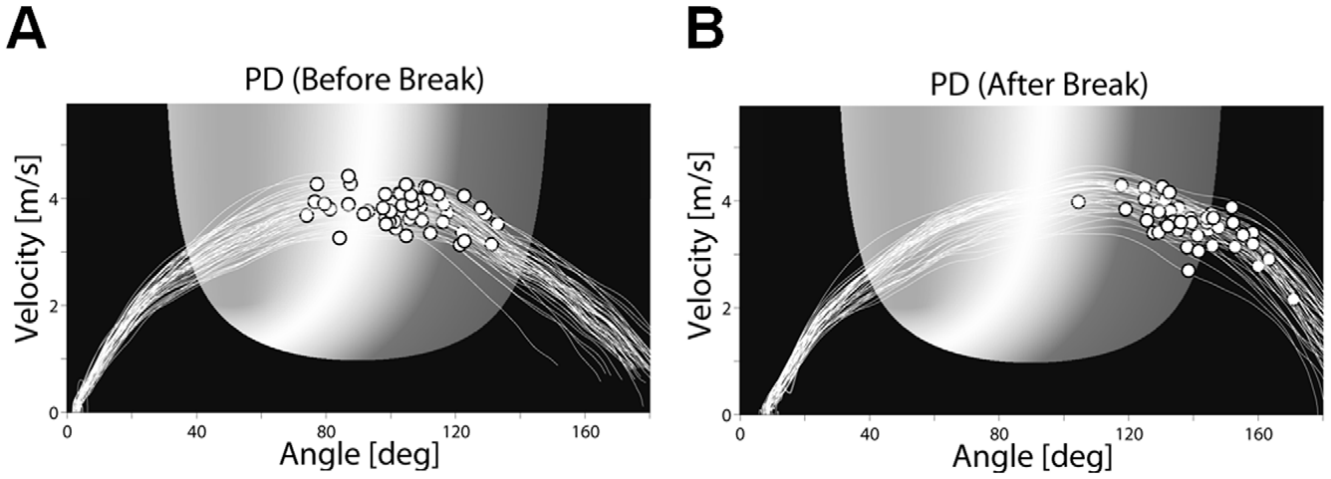
Sample data of release control of a PD patient. Throwing trajectories and moments of ball release of a PD patient before (**A**) and after (**B**) a break. Movement direction is from left to right. Note that although there is no time dimension in this figure, when comparing A to B, the release points in B (with higher release angles) could only be achieved by releasing later relative to A (see File S1).

**Figure 7:**
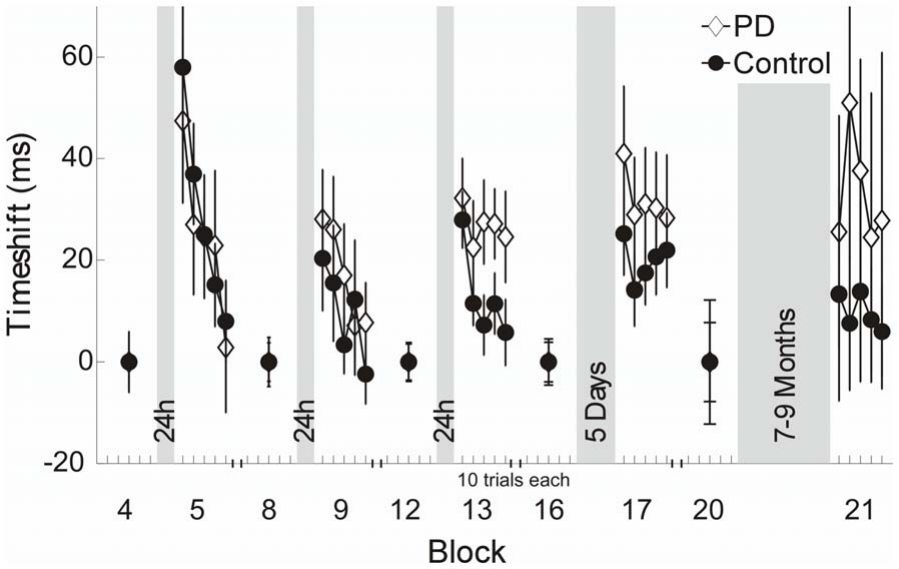
Group averaged *timeshift* measure quantifying release control. Average *timeshift* for the last block of a session and the first block of the subsequent session for patients and the control group. Note that *timeshift* values after breaks are displayed in five series of ten trials to illustrate change with continued practice. Error bars denote standard error of the mean.

Figure 6 shows sample data of throwing trajectories and release points of a PD patient before and after the last break of 24 h. Release is clearly delayed after the break, resulting in poorer performance (darker areas on the solution manifold). Group results of the *timeshift* analysis are illustrated in Figure 7. Average *timeshift* values of the first 10 trials of each new session increased in both groups with respect to the reference of 50 trials before rest, meaning that release was delayed in both groups (PD: *M* = 38.1 ms, *SD* = 30.9 ms; Control: *M* = 31.2 ms, *SD* = 29.5 ms). Within each first five series after rest, *timeshift* values in both groups decreased again, i.e. timing became better. This was supported by a significant effect for series in the 2 (group)×4 (rest)×5 (series) ANOVA with repeated measures (*F*(1.8,53.9) = 10.9, *p*<.001, *η_p_^2^* = .27). In addition, we found a significant rest×series interaction (*F*(5.3,159.4) = 2.4, *p*<.05, *η_p_^2^* = .08), which was due to the different change in *timeshift* between the first post-rest session and the other three. There were no significant group or rest×group interaction effects (group: *F*(1,30) = 2.0, *p* = .17, *η_p_^2^* = .06; rest×group: *F*(2.6,77.5) = .5, *p* = .66, *η_p_^2^* = .02), indicating that both groups released later at the beginning of each new practice session compared to the end of the previous sessions. However, leaving out the first post-rest session because of its discrepancy from the other sessions, an additional 2 (group)×3 (rest)×5 (series) ANOVA indicated a tendency for a group difference in timing (*F*(1,30) = 3.7, *p* = .06, *η_p_^2^* = .11).

Similarly, *timeshift* increased, on average, from the last practice block to the first long-term retention session block in both groups, with a greater mean increase in PD patients. However, no significant main or interaction effects were found. Looking more closely at the two patients with performance decreases in long-term retention, revealed a delayed release for patient 16 (*M* = 42.29 ms, *SD* = 16.4 ms). Throwing trajectories of patient 25 did not satisfy the conditions for analysis.

In summary, after rest, timing of release was delayed in both groups. There was a tendency that the delay was greater in patients relative to controls from the second post-rest session on.

## Discussion

The goal of this study was to examine the specific problems of PD patients in different stages of motor skill learning and the influence of typical parkinsonian control deficits on the learning outcome. In reference to studies about the role of BG in motor learning and patient studies, it can be expected for de novo motor learning that non-demented, mild to moderate PD patients on medication should have no problems in early learning stages but should show deficits in fine-tuning of a newly practiced skill. In addition, there are indications that motor control deficits might influence retention performance of PD patients. We will discuss our results with respect to consistencies and inconsistencies with these expectations.

### Expectation 1: Early learning is intact in PD patients

We showed that the PD patients were able to improve in a novel virtual throwing task. Under the assumption that motor learning is represented by performance changes as a function of the amount of practice, patients improved similar to healthy subjects over the course of five practice days. However, performance of the patients remained below that of the control group. The rate of performance improvement on the first day was equally high for the PD patients compared to the healthy subjects. Improvements at that early stage resulted from an exploration of the task solutions and tolerant areas, as represented by the component *Tolerance* (*T*). With reference to findings documenting that it is primarily the cerebellum that is involved in early learning stages of a novel motor task [24]–[26], [3], our results confirm the expectation that PD patients are not impaired in this phase. Reasons could be that they compensate the BG failure with activation of other brain structures, like the cerebellum [41], [42].

*Consistent with expectation: We found intact learning of a new motor skill at an early stage in PD patients.*

### Expectation 2: Fine-tuning is impaired in PD patients

Expectation 2 originates from the findings of few studies addressing late learning in PD where late learning can be determined by the amount of practice [10], [13]. However, the identification of the fine-tuning stage simply by the amount of practice is oftentimes difficult. In our data this becomes especially evident regarding the constant performance decrements at the beginning of new sessions in the patient group. Analyzing the performance score, we found that patients overcame these initial deficits and even exceeded their previous performance level. Hence, they learned more each day instead of only regaining what they had already achieved in the first day. To be able to determine to what extent, however, this learning can be ascribed to fine-tuning and to what extent early learning components, because of the break, might be involved, we need an additional approach to parse the behavioral results from the internal mechanisms that underlie them.

Earlier, we defined fine-tuning as selection and reinforcement of appropriate motor commands, as well as inhibition of unwanted perturbing commands, i.e. an improvement of the signal-to-noise-ratio. There are studies reporting higher movement variability in PD patients [43], [44] which they cannot reduce as well as healthy people [19], i.e. fine-tuning seems to be poorer in PD patients. However, variability is not only caused by higher motor noise but also through task exploration and the utilization of covariation between execution variables. To be more precise, what people seek in order to enhance task accuracy is the reduction of motor noise, not variability per se. Therefore, SD of execution variables is not appropriate to detect motor noise and reduction of it. In the TNC-method *Noise Reduction* (*N*) is determined after having eliminated the influence of *Covariation (C)* and *Tolerance* (*T*) [37]. Hence, *N* can ascribe performance improvements solely to a reduction of motor noise, and a better attuning of execution variables is quantified by *C*. Thus, the contribution of *N* and *C* to performance change over practice indicates to what extent the change is accomplished through fine-tuning. In our results, *N* did not show any significant differences between PD patients and control subjects. Furthermore, the contribution of *N* to performance changes increased continuously throughout practice in both groups, meaning that both groups were similarly good at reducing motor noise to enhance their performance. Importantly, this also implies that the patients did not decrease in performance after rest due to higher motor noise nor did the overcoming of this decrement within a practice session arise from an enhanced reduction of motor noise.

The component *Covariation* had no effect in neither of the groups. This was not unexpected since the contribution of *C* is highly dependent on the task and the position of the target, in other words on the shape of the solution manifold that changes with different target locations [37]. For target positions that result in a more vertical solution manifold *C* seems to contribute less to performance changes even in healthy young participants [37], [38].

*Inconsistent with expectation: We could not find impairments in the fine-tuning process in the PD patients. The inconsistency might be due to different noise measures (execution level vs. result level). Moreover, studies that find impairment in fine-tuning, find it only for advanced disease stages* [*10*] *, for patients “off” medication, or motor control symptoms might have influenced the result* [*11*] *.*

### Expectation 3: Retention performance in PD patients is influenced by motor control deficits

In the motor learning context, retention usually stands for the preservation of a movement specific skill over a period of rest. In Skittles, this would include adequate generation of the movement trajectory and timing of the release. The constant performance decrease in the patient group in each first block of a new session might suggest that PD patients had problems retaining this previously acquired skill for at least 24 hours. The performance decrease was due to the component *Tolerance*, i.e. patients started off throwing in less tolerant regions and hence achieved fewer points. This could indicate that they failed to properly store the task model and had to reacquire it each day. However, especially release timing is, due to its relatively short time window, sensitive to random changes in neuronal processing. Control deficits like movement initiation problems in PD can be a cause of such changes. The timing analysis demonstrated that both experimental groups constantly released later after rest compared to before rest, as represented by the higher *timeshift* values. Aside from the first post-rest session, the delay was generally higher in patients than in controls and it had a negative effect on their performance. This is because, according to Figures 1C and 6, if participants release later, they land in less error tolerant areas.

Since poorer retention of release timing would be expected to cause later as well as earlier release times after rest compared to before rest, the constant release delay indicates that the post-rest performance decrements in the patient group were not due to retention deficits.

Performance of the control group decreased in the first block of the first post-rest session (see Fig. 3, session 2, block 5) where they also showed the greatest increase in *timeshift*, i.e. poorest timing (see Fig. 7). In contrast to the patients, however, their performance did not continue to decrease after rest in the following sessions, due to an improved timing. Apparently, healthy elderly present similar timing problems as PD patients but they can control them better in the course of practice. Considering the symptomatic problems of hypokinesia and related movement initiation deficits in PD, poorer timing and hence lower performance after rest could have arisen from constant impairment of initiation of ball release at the beginning of practice as opposed to retention deficits.

The results of the long-term retention session further support that impaired release initiation might have diminished post-rest performance in PD. Four out of six PD patients were able to retain their performance in the Skittles task equally well as the control group over seven to nine months. Examining the *timeshift* for the two patients with less good post-rest performance revealed delayed release timing for one patient. The performance of the other patient did not satisfy the conditions for the *timeshift* analysis. His trajectories in the retention session differed severely from the last block of the five practice days, indicating either a progression in disease and cognitive problems, respectively. Even though the results of only four PD patients are not representative, it is striking that those patients could retain the Skittles task over a period of up to nine months equally well as the control subjects.

Furthermore, all patients improved after the initial post-rest decrements in the five practice sessions and they even exceeded their pre-rest level. This was also related to release timing. Concretely, timing improved in the first 50 trials of each session (*timeshift* approached zero in Fig. 7) and according to the results of the *T* component, patients therewith threw in more tolerant areas again (Fig. 4B). This indicates that impaired release initiation is not an irreversible symptom. Other studies also find that PD patients can improve their control deficits with practice [29], [33].

*Consistent with expectation: Poorer performance in short- and long-term retention tests might be due to problems in movement initiation at the beginning of a new session rather than due to poorer retention.*

The interpretation about influences of movement initiation in PD on motor learning is still speculative at this point. Often, no significant correlations are found between motor function and learning ability. However, these correlations are usually done with the UPDRS motor score that combines all motor symptoms related with PD. Given that some deviant results between studies can possibly be explained by influences of very specific control problems [33], [12], [10], [19], a more systematic examination of convolutions between learning and control needs to be considered. Our *timeshift* analysis is in that sense more specific, connecting performance changes only to one symptom, namely impaired movement initiation. But, further experiments are necessary to specifically scrutinize this matter.

### Conclusion and Implications

In conclusion, our results confirm that non-demented, medicated patients with mild to moderate PD can improve their performance in a novel motor task and that they show no impairments at an early learning stage. However, we did not find impairments in fine-tuning nor in retention. Performance decreases after a break or stagnations during practice might rather be confounded with motor control deficits which can recover in the course of practice. This is important for therapeutic implications. Knowledge about these problems will be very helpful in motivating patients to keep practicing despite initial difficulties and to prevent frustration.

## Supporting Information

File S1 **Quantification of release timing using a numerical procedure.**
(PDF)
