# Supplementary material for: Motor Skill Learning, Retention, and Control Deficits in Parkinson's Disease
Source: PLoS One. 2011 Jul 8;6(7):e21669. doi: 10.1371/journal.pone.0021669 (PMC3132742; doi:10.1371/journal.pone.0021669)
Supplement: File S1 — Quantification of release timing using a numerical procedure. (PDF) [file pone.0021669.s001.pdf]

Quantification of release timing using a numerical procedure

Assuming that after an exploration phase participants produce relatively stable trajectories over practice and only the release changes "over time", release timing can be quantified using a numerical optimization procedure that aligns the angle time profiles of trials and allows assessing release timing variability.

We tested the assumption of stable trajectories within subjects by determining variances between angle time profiles of trials within subjects over the 20 practice blocks. The measure for variance was the root mean square error (RMSE) between angle profiles. As a result, RMSE reduced significantly after the third block and remained relatively constant until the end of practice. In addition, RMSE between trials within subjects was about 60% lower than a constellation of angle profiles randomly drawn from all subjects. Hence, subjects vary interindividually in their throwing manner, but intraindividually they are relatively stable.

The residual variance between angle profiles of different trials within subjects can be explained with variance in throwing trajectory, in particular changes in angle, or with variance in timing. We tested both alternatives and found a higher explanatory power of the timing hypothesis. Statistical tests secured this with a clear tendency. Thus, with reference to the rationale that, especially in PD, timing is more sensitive to control deficits than the generation of throwing trajectories, the *timeshift* analysis can be applied to quantify release timing.

In the following, the quantification procedure is explained by example of three sample trials (n = 3). Within the data collection procedure angle time profiles of trials were synchronized at the point of release (t = 0) (Fig. S1A). For a window of 300ms around release, the angle profiles were shifted in time, with decremental steps starting from shift value s_{start} = 10ms, to reduce pairwise distances. To minimize

computational effort, coefficients of third order polynomials of the angle time profiles were determined using the function polyfit in MATLAB®, and the coefficients of each trial were shifted.

A custom-made algorithm in MATLAB® was used for the following stepwise optimization procedure:

1. Shift trial *a* by the current time shift value s_i (Fig. S1B).
2. After each single shift, calculate the total root mean square error ($RMSE_t$) between all polynomials (Fig. 1C) and repeatedly shift trial *a* with shift value s_i until $RMSE_t$ does not reduce further. For the three sample trials, $RMSE_t$ is computed as follows:

$$RMSE_t = RMSE_{a'-b} + RMSE_{a'-c} + RMSE_{b-c} \quad (1)$$

   where $RMSE_{a'-b}$ equals the RMSE between trials a' and b, $RMSE_{a'-c}$ is the RMSE between a' and c, and $RMSE_{b-c}$ represents the RMSE between b and c.
3. Repeat step 1 and 2 for all other trials. I.e. in our three trial example, trial *b* and after that *c* are shifted by shift value s_i until $RMSE_t$ does not reduce further.
4. Change the direction (positive vs. negative) of the shift value:

$$s_i = s_i * (-1) \quad (2)$$

5. Repeat steps 1-4 n times.
6. Change shift value direction and half the value size:

$$s_i = (s_i * (-1)) / 2 \quad (3)$$

7. Repeat steps 1-6 until the shift value is less than 0.1 ms.

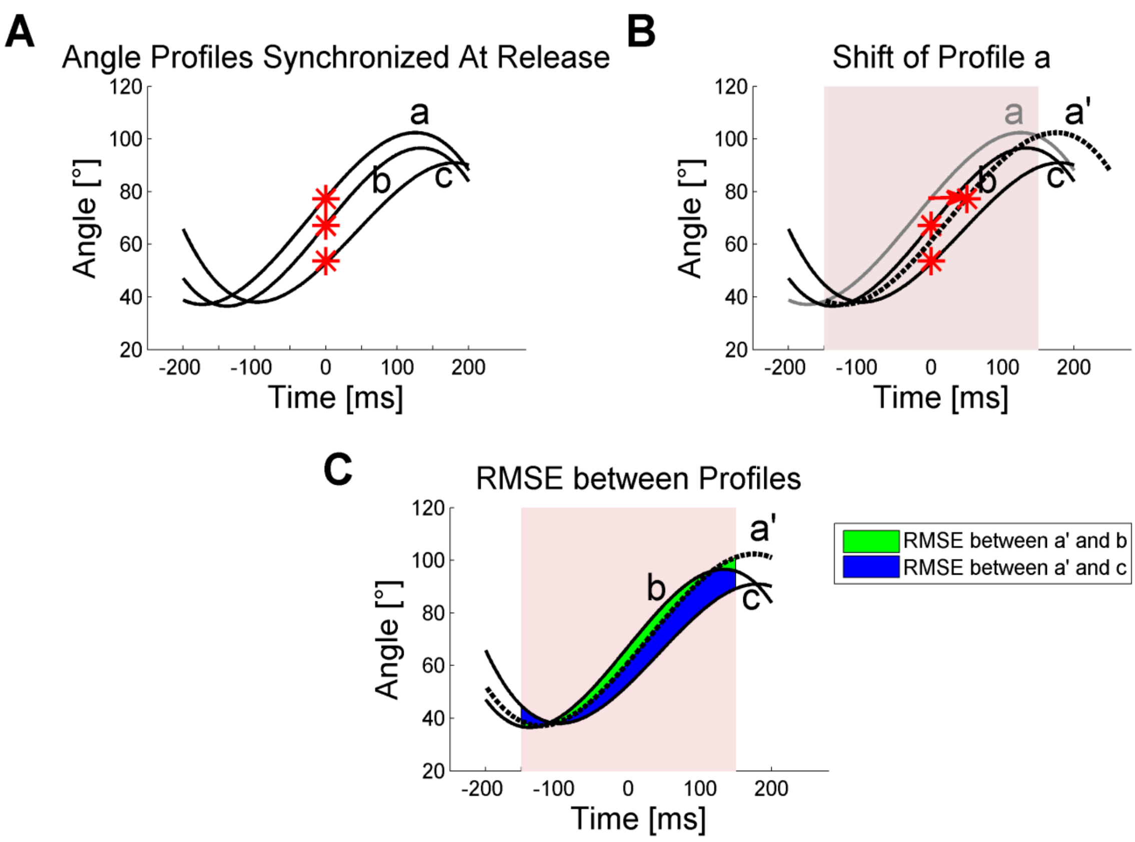

Fig. S1. Schematic illustration of the *timeshift* quantification. **A:** Angle profiles of 3 trials (a, b, c) are plotted with their release times (red stars) synchronized at time t = 0. **B:** The alignment procedure shifts profile *a* by 50ms to profile *a'*. For clarification a large shift value was used here. Note that in the procedure, the first shift value is 10ms. The shift and the following RMSE calculation were done for 300ms around release. **C:** RMSE between trials. Note that for reasons of illustration, only areas between *a'* and *b* as well as *a'* and *c* for RMSE determination are displayed. RMSE between *b* and *c* was also calculated and all three RMSE determined total $RMSE_t$.

This optimization procedure can move the release point of each trial backwards or forwards in time resulting in the *timeshift* measure in ms (Fig. S2). *Timeshift* is positive when the release is delayed and negative when the release is early. The procedure can be applied to compare timing between individual trials as well as sets of trials. When two sets of trials are compared, trials of both sets are passed to the

algorithm at once and angle time profiles are collectively aligned. After the procedure, timing information (*timeshift*) for all trials of set 1 are averaged and all trials of set 2 are averaged to be compared against each other.

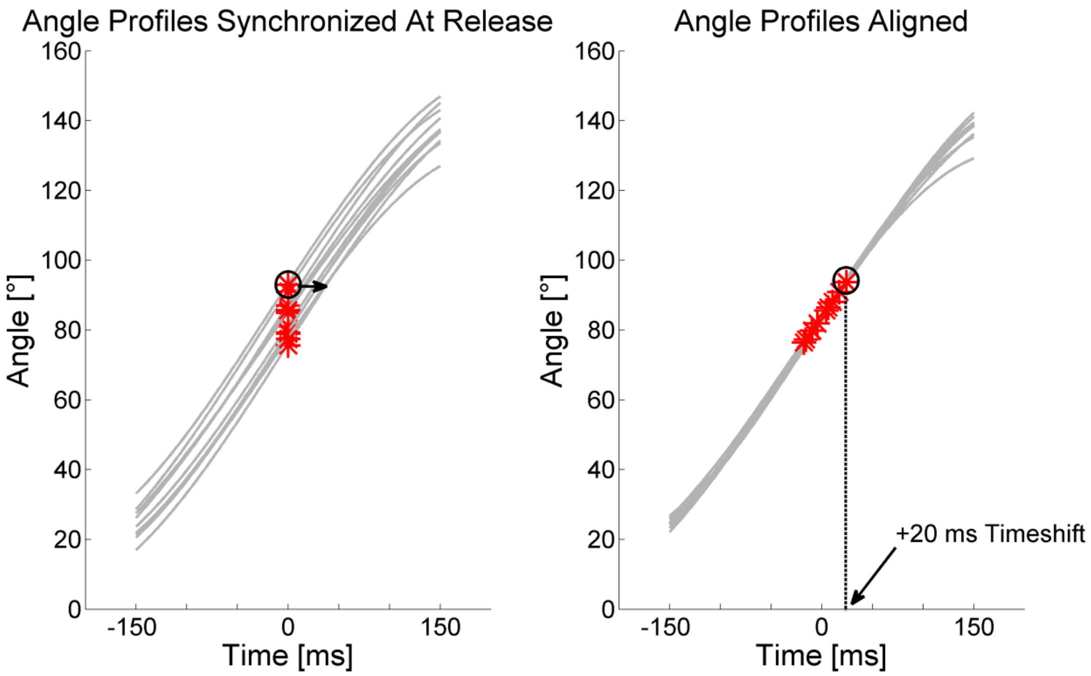

Fig. S2. Illustration of the *timeshift* measure. **A:** Angle profiles of 10 trials are plotted for a window of 300ms around release and with their moments of release synchronized at time t = 0. The alignment procedure shifts angle profiles back and forth in time to reduce RMSE between the profiles. **B:** Result of alignment optimization. Release points are positive or negative in the time dimension which equals the *timeshift* values. Encircled is one release point shifted by 20ms by the procedure.
